# *Magellon* – an extensible platform for cryo-EM data visualization, management and processing

**DOI:** 10.1107/S2052252525007432

**Published:** 2025-09-05

**Authors:** Behdad Khoshbin, Puneeth Damodar, Rupali R. Garje, Frank H. Schotanus, Nandagopal Nair, Lai Wei, Jan-Hannes Schäfer, Gabriel C. Lander, Michael A. Cianfrocco, Scott M. Stagg

**Affiliations:** a Institute of Molecular Biophysics Florida State University Tallahassee FL USA; b Department of Computer Science Florida State University Tallahassee FL USA; c Life Sciences Institute University of Michigan Ann Arbor MI USA; d Department of Integrative Structural and Computational Biology Scripps Research La Jolla CA USA; e Department of Biological Chemistry University of Michigan Ann Arbor MI USA; f Department of Biological Science Florida State University Tallahassee FL USA; Boston University School of Medicine, USA

**Keywords:** *Magellon*, automation, data processing, cryo-EM, structural biology, macromolecular structures

## Abstract

Single-particle cryo-electron microscopy (cryo-EM) has revolutionized structural biology by enabling high-resolution determination of macromolecular structures. However, the field faces challenges in data management, processing workflow integration and software extensibility. We present *Magellon*, an innovative cryo-EM software platform that addresses these challenges through a modern microservices architecture. *Magellon* consists of an extensible backend with a web based frontend that we call *Magellon Viewer*. Together, these combine high-performance computing capabilities with an intuitive user interface, enabling researchers to efficiently process and analyze cryo-EM data. The platform’s distinguishing features include a plugin based architecture, distributed processing capabilities, comprehensive monitoring systems, and a novel approach to data organization and visualization. A key philosophy of the approach is that the *Magellon* backend provides a platform that uses robust industry-standard libraries to orchestrate computational tasks while offering users and developers flexibility in selecting the computational resources for performing calculations. *Magellon* represents a significant advancement in cryo-EM software infrastructure, offering flexibility, scalability and extensibility while maintaining ease of use.

## Abstract 2

This work presents a new cyberinfrastructure framework for cryo-EM structure determination, *Magellon*, which offers a robust and flexible approach to cryo-EM data management. We show the power of this platform by building a frontend web interface, *Magellon Viewer*, on top of *Magellon*.

## 1. Introduction

### 1.1. Cryo-EM and automation

Automation has played a pivotal role in the explosive growth of single-particle cryogenic electron microscopy (cryo-EM) and the accessibility of the technique to investigators who are not experts in electron microscopy. Automated tools and software can be found in all stages of cryo-EM structure determination, including specimen preparation (Darrow *et al.*, 2019 ▸; Esfahani *et al.*, 2024 ▸), data collection (Suloway *et al.*, 2005 ▸; Mastronarde, 2005 ▸) and image analysis ( *et al.*, 2009 ▸; Kimanius *et al.*, 2021 ▸; Punjani *et al.*, 2017 ▸; Rosa-Trevín *et al.*, 2016 ▸; Grant *et al.*, 2018 ▸). The development and implementation of such tools has had a transformative impact on the field of structural biology, as they render the complex process of single-particle cryo-EM structure determination more tractable, repeatable and systematic.

The increased use of cryo-EM structure determination has been accompanied by an expanded collection of academic software for data processing, which continues to grow (Cheng & Yu, 2024 ▸; Mendez *et al.*, 2023 ▸; Li *et al.*, 2025 ▸). This growth has been accelerated by the rise of machine learning in computer vision, which has been integrated into nearly all aspects of the cryo-EM image analysis pipeline (Bepler *et al.*, 2019 ▸; Li *et al.*, 2020 ▸; Jamali *et al.*, 2024 ▸; Wagner *et al.*, 2019 ▸). The software packages developed to address the various steps of data processing can be classified into three primary types: (1) comprehensive, all-in-one packages such as *cryoSPARC* (Punjani *et al.*, 2017 ▸) or *EMAN2* (Tang *et al.*, 2007 ▸), where each processing step has been coded individually in the software package environment; (2) hybrid packages, such as *Scipion* (de la Rosa-Trevín *et al.*, 2016 ▸), *Relion* (Scheres, 2012 ▸), *Appion* (Lander *et al.*, 2009 ▸) or *cisTEM* (Grant *et al.*, 2018 ▸), which integrate both native algorithms and external software through wrapper interfaces; and (3) customized, bespoke data processing pipelines, where individual investigators install the stand-alone programs and write convenience scripts to shuttle data between them. The advantage of the all-in-one packages is that they are relatively easy to install and learn to use, but have limited flexibility and extensibility. In contrast, bespoke pipelines, while highly customizable, can be complex to set up and difficult to manage. Hybrid software packages aim to combine the advantages of both approaches by providing a standardized interface that integrates both native algorithms and external software.

Hybrid systems and bespoke pipelines face several technical architecture challenges that affect workflow efficiency and the longevity of the software. One major problem is the complexity of software integration, as these systems often struggle to seamlessly integrate multiple processing tools and algorithms. Within the same vein are issues with data organization – when databases are incorporated into a software package, there is usually a lack of robust methods for portable data organization, which creates problems for inter-facility transfers. Scalability constitutes an additional limitation, with systems often unable to smoothly scale from personal workstations to large computing clusters. Finally, introducing new functionality can present considerable barriers, as integrating new software or algorithms typically demands comprehensive knowledge of the underlying codebase.

We present here a new hybrid software package, *Magellon*, an extensible platform designed to enable intuitive, point-and-click data viewing, organization and analysis. Notably, the platform is open source, flexible and designed for cross-compatibility across different operating systems. *Magellon* employs a microservices architecture to enable independent scaling of components, while its plugin based design facilitates seamless integration of new algorithms without modifying core code. Considerable effort was devoted to the design of *Magellon*’s backend, making it deployable and consistent across different computing environments, including Windows, Linux and MacOS. Additionally, *Magellon* employs industry-standard libraries and modern protocols to enable installation on personal laptops while maintaining scalability to data-center-sized infrastructure. As a demonstration of *Magellon*’s capabilities, we developed the *Magellon Viewer* – a web based, interactive frontend built with *React JavaScript* that leverages the *Magellon* backend. The platform’s modular design enables research teams to customize or replace components, such as the *Viewer*, with specialized interfaces tailored to specific needs, or even develop alternative frontends for particular applications. With potential for future expansion through micro-frontends, *Magellon* is positioned to become a critical component of cryo-EM cyberinfrastructure, supporting both individual researchers and large research groups.

## 2. Design

### 2.1. Design principles

The foundational philosophy behind *Magellon* is that it should be a platform-as-a-service (PaaS) tool for cryo-EM data processing and algorithm deployment. The architectural core of *Magellon* is structured to handle database organization, job submission and user accounts. Additionally, *Magellon* offers extensible tool implementation through a well defined plugin/toolbox interface in addition to enabling portability for data import/export. The combination of these tools establishes a foundational platform that can be used for data viewing and processing in a web-tool named the *Magellon Viewer*. This design ensures that training and maintenance remain manageable while promoting extensibility, thereby enabling the broader scientific community to contribute features and enhancements so that development is not limited to the core *Magellon* developers.

### 2.2. *Magellon* backend

**Figure 1:**
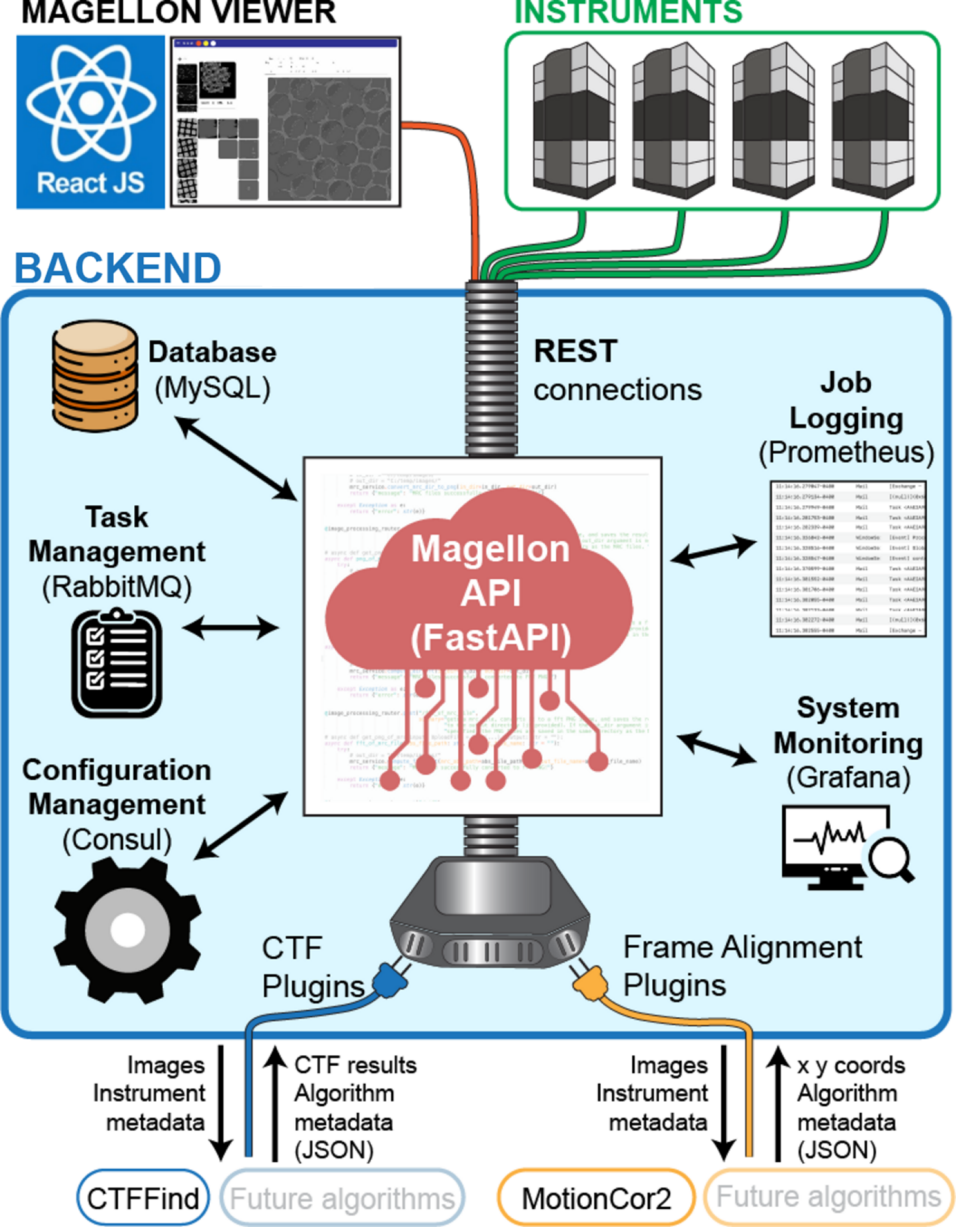
*Magellon REST* computational architecture and API framework. The heart of *Magellon* is the *Magellon* API which utilizes industry-standard libraries and frameworks that altogether form the *Magellon* backend. The *Magellon Viewer* frontend and plugins connect to the backend through standardized outlets that enable extensibility of the platform.

The *Magellon* backend comprises a central data orchestration framework and a set of plugins, functioning similarly to a microservices environment where each component operates independently (Fig. 1 ▸). This design offers flexibility, scalability and maintainability. The backend manages primary tasks, schedules jobs and handles communication between plugins, while the plugins encapsulate specific algorithms that function as independent services. *Magellon* was designed to use resources available either on the host computer or across a network by leveraging representational state transfer (*REST*) programming. Using the *REST* framework, tasks are allocated to different workers that can reside on any computer accessible over the network with the *Magellon* software installed. To further the robustness of our backend infrastructure and accommodate ease-of-use for developers aiming to extend or interact with *Magellon* tasks, we developed a *Magellon* application programming interface (API). The *REST* API framework was written using *FastAPI* (https://fastapi.tiangolo.com) and *Swagger* (https://swagger.io) (Fig. 1 ▸), and enables task requests to be transmitted over a network. *FastAPI* provides for separation of controllers for each purpose (*e.g.* separate controllers web, database, processing *etc*.) and enhances code organization and readability, while *Swagger* provides an interface for API documentation and testing.

**Figure 2:**
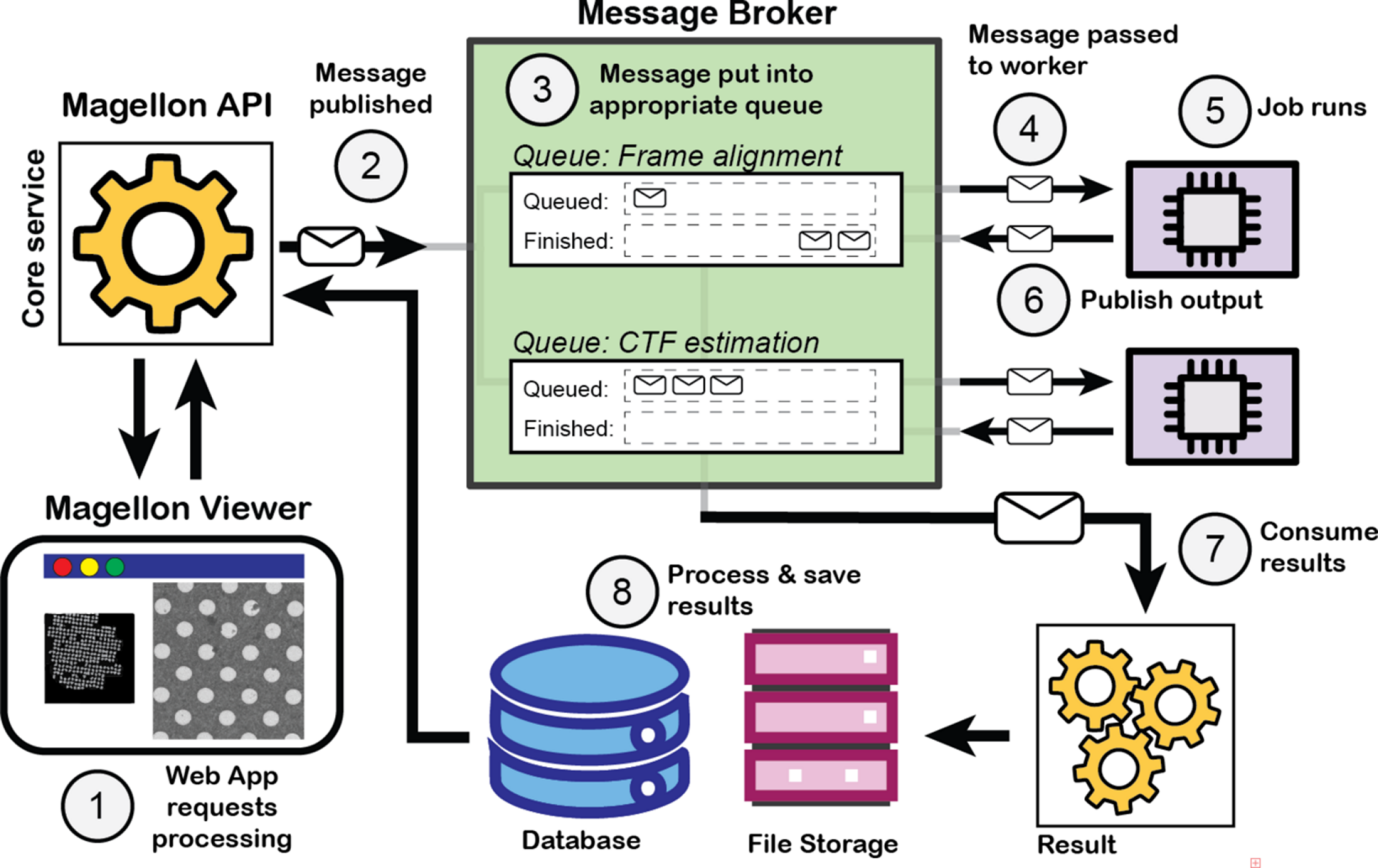
Task management in the *Magellon* backend. (1) Tasks are initiated from the frontend and passed to the *Magellon* API, (2) messages are published to the *RabbitMQ* message broker. (3) Messages (*i.e.* job requests) are placed in a job queue. (4) Jobs are passed to a data processing worker, where (5) the job runs. (6) When the job completes the outputs are published back to *RabbitMQ*. (7) *RabbitMQ* routes the results to the results processor that (8) inserts metadata into the *Magellon* database and saves the results to the appropriate paths in the file system and notifies the *Magellon* API of completion.

To effectively manage the diverse processing tasks that could be carried out within the *Magellon* interface, which could range from seconds to hours of computational time, a robust task manager was implemented using *RabbitMQ* (https://www.rabbitmq.com) for message queuing and management (Fig. 2 ▸). *RabbitMQ* is an open-source message broker with established reliability and efficiency in handling asynchronous communication between distributed systems. *RabbitMQ* serves as the central orchestrator for *Magellon* processing while the *FastAPI* backend serves as the central coordinator, managing task distribution, monitoring execution status across the distributed system and triggering dependent processing steps based on task completion. This computational organization structure ensures that tasks are efficiently scheduled, queued and executed across various computational resources, optimizing *Magellon*’s performance and scalability.

The *Magellon RabbitMQ* implementation includes systematic error logging, retry mechanisms with exponential backoff and failure escalation protocols. Failed tasks are automatically logged with detailed error messages, and the system supports configurable retry policies with dead letter queues for permanently failed tasks. When plugins fail, the architecture ensures that failures remain isolated and do not propagate system-wide. The queue management prevents data loss and maintains processing continuity. An advantage of this approach is that data processing is very robust against processing interruptions; the whole system can be restarted and the processing will continue where it left off.

Maintaining consistent and validated data exchange between different *REST* clients is crucial to *Magellon*’s efficiency and the integrity of analyses. To this end, *Magellon* employs *Pydantic* models (https://docs.pydantic.dev) for data structuring and validation, including the consistent serialization and deserialization of messages as *Pydantic*-validated *JSON*. *Pydantic* is a Python library designed for data parsing and validation that integrates seamlessly with *FastAPI* and *Swagger* frameworks. *Pydantic* ensures that incoming data adheres to the defined schemas and formatting requirements, thereby minimizing the risk of errors occurring during processing interactions. For example, in *Magellon* there is a *Pydantic* model for images (*ImageDtoBase*) that defines all of the metadata and types (*e.g.* stage position, image shift, spot size, beam intensity, pixel size *etc.*) that are expected for a TEM image being imported. Thus, data import is standardized regardless of what instrument or software is used for data acquisition. By standardizing data formats in this manner, *Magellon* facilitates streamlined interactions within the platform, simplifying and standardizing the development process.

### 2.3. Frontend: *Magellon Viewer*

The *Magellon Viewer* is entirely web based to maximize accessibility, ease of interaction, and exposure of users to their data and metadata while simultaneously leveraging the suite of backend *Magellon* tools. The web navigation is designed to be intuitive, being primarily image based with a hierarchical organization of the low- and high-magnification images that comprise a dataset. Relevant metadata are prominently displayed and accessible, ensuring that users can access any associated information with a dataset (*e.g.* microscope and detector settings). Web pages are generated dynamically, and interactivity is achieved through *JavaScript* using the *React* framework (https://react.dev), which ensures a responsive and seamless user experience.

Interaction between the frontend and backend is facilitated through *RESTful* API calls. When a user interacts with the web interface, such as by selecting a dataset or processing task, the frontend sends requests to the backend via the *Magellon* API. Processed data are communicated to the frontend, updating the interface in real time to enable navigation of large datasets, track the status of processing jobs and review results instantly. Associated metadata are displayed in a structured and user-friendly format, often alongside corresponding images. For instance, metadata such as acquisition parameters (*e.g.* kiloelectronvolts, magnification) and processing results [*e.g.* particle selection, contrast transfer function (CTF) estimation] are integrated into the display. The metadata are dynamically loaded from the backend, ensuring that any updates or changes made during data processing are immediately reflected in the interface, which is particularly important for users to make informed, real-time decisions based on the incoming data.

### 2.4. *Magellon* database structure

To handle the complexity of metadata associated with a cryo-EM data collection experiment, *Magellon* employs the relational database *MySQL* (https://www.mysql.com) for efficient storage and retrieval. *Magellon* database interactions utilize the *SQLAlchemy* Python libraray with *Pydantic* models for validation and serialization. Many concepts were taken from the database architecture developed previously for the data acquisition software *Leginon* (Suloway *et al.*, 2005 ▸), but the *Magellon* database is greatly simplified to facilitate easier data export and transfer between data collection sites. A potential problem in designing a database where the metadata can be transferred between different physical locations is that a mechanism is needed to ensure that primary keys are not duplicated. Thus, to avoid primary key conflicts, we use universally unique identifiers (UUIDs) as primary keys. This guarantees that all systems have globally unique IDs for their image records, allowing different databases to be merged readily into one database without concerns about conflicts. There is no built-in UUID column type in *MySQL*, thus UUIDs (UUID version 4 – random) are stored as binary(16) by removing four characters and storing each two hexadecimal numbers in a single byte. The full database schema are available for viewing on the *Magellon* website (https://magellon.org).

### 2.5. *Magellon* adaptors to enable cross-software compatibility

**Figure 3:**
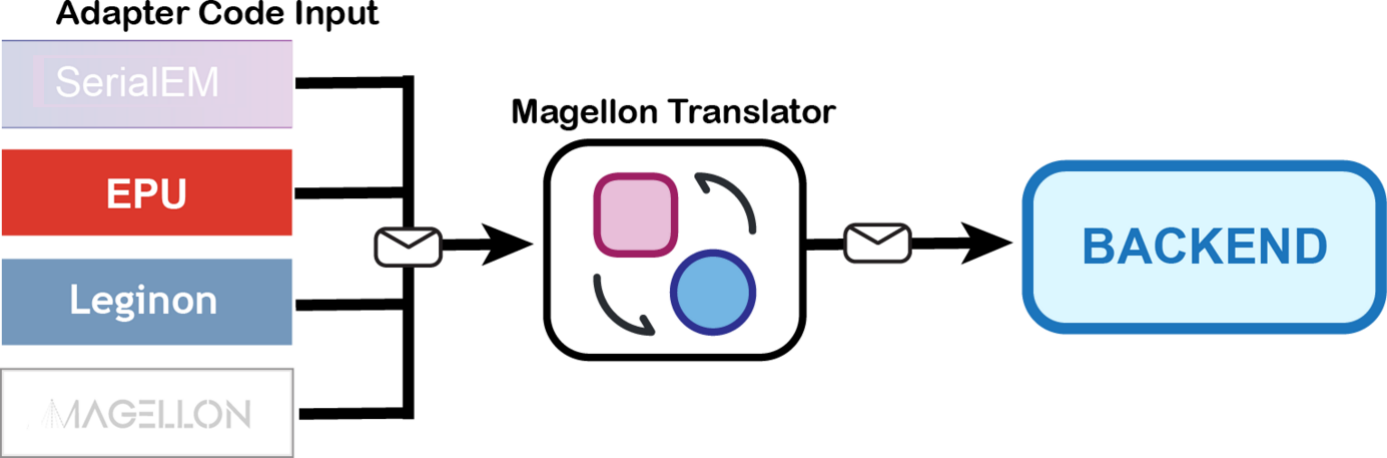
Adapter code unifies metadata from multiple different input sources and inserts the data in a common format into the *Magellon* database.

*Magellon* was developed to be agnostic to the software used for data collection. Data and metadata are ingested and assigned to unified metadata fields so that the import process is the same for all data collection packages. This approach is accomplished with a *Magellon* translator layer that populates defined metadata fields and inserts them into the *Magellon* database. Pieces of code, called ‘Adapters’, read metadata from different data collection packages and interface with the translator to enable imports from various software sources. Adapters have been written for the data collection software packages *Leginon* (Suloway *et al.*, 2005 ▸) and *Thermo Fisher Scientific EPU*, and adapters for *SerialEM* (Mastronarde, 2005 ▸) and *Gatan Latitude* are currently under development (Fig. 3 ▸). Additionally, *Magellon* import/export utilities have been written to enable data transfer between different sites.

### 2.6. *Magellon*’s plugin interface offers flexible incorporation of software tools

*Magellon* is designed with an open architecture that promotes extensibility, facilitated by a robust plugin system that streamlines the testing and integration of new algorithms [*e.g.* CTF, fast Fourier transforms (FFTs), particle picking, frame alignment *etc.*]. Built on a microservices architecture, each data processing algorithm exists as a distinct plugin or microservice, offering significant advantages over monolithic designs. Plugins can be independently developed, tested, published and shared, while also being deployable on separate systems or via containers with isolated dependencies. Each plugin functions as a self-contained *FastAPI* project encapsulating a single algorithm, ensuring modularity and independent development cycles. This approach not only enhances scalability but also simplifies deployment through containerization technologies like *Docker* and orchestration tools like *Kubernetes* (https://kubernetes.io).

*Magellon*’s plugin architecture draws inspiration from the *Akka* framework’s (https://akka.io) actor model, where each plugin operates as an independent actor within the system. Plugins communicate with the backend exclusively through message-passing via *RabbitMQ*, maintaining dedicated message queues that enable asynchronous task processing, state isolation and operation encapsulation. This approach offers significant advantages: it prevents individual plugin failures from affecting the entire system; hierarchical supervision automatically restarts failed plugins; and state preservation mechanisms safeguard data during interruptions. The design ensures location transparency, allowing plugins to be relocated or scaled without system disruption. By utilizing message-passing for parallelization, the architecture efficiently manages resources and minimizes bottlenecks while enabling horizontal scaling and dynamic resource allocation. Further, each plugin’s isolated memory space ensures optimal memory management and reduces conflicts. These architectural choices make *Magellon*’s plugin system inherently extensible, scalable and resilient, enabling cryo-EM algorithm developers in the community to deploy their specialized algorithms as *Magellon* plugins without concerning themselves with peripheral challenges like data management, system control, pipeline configuration or parallelization. Developers can thus focus exclusively on algorithm development while *Magellon* handles the infrastructure.

### 2.7. Flexible *Magellon* deployment for individual users or large facilities

*Magellon* was designed with a ‘deploy anywhere’ philosophy, emphasizing ease of installation while maintaining flexibility for diverse computational environments. This approach directly addresses the software compatibility and installation challenges commonly faced in hybrid computing infrastructure projects. The deployment architecture supports both simple single-machine installations for individual researchers and complex distributed setups for large facilities, achieved through two complementary approaches: *Docker Compose* for containerization and a custom command-line interface (CLI) for management.

*Magellon*’s deployment architecture accommodates three primary scenarios: single-machine, distributed and hybrid deployment. In the single-machine deployment regime, all components of *Magellon* run on one computer while maintaining isolation through containerization, requiring minimal configuration yet providing full platform functionality. For distributed deployment, *Magellon* components can be installed across multiple networked computers, with each component placed strategically to utilize specialized hardware (such as GPU clusters) while maintaining seamless system-wide communication. In hybrid deployment installations, *Magellon* can bridge local resources with cloud infrastructure, allowing researchers to maintain core functions locally while dynamically expanding processing capabilities to external resources as needed. This flexibility ensures that *Magellon* can adapt to computational needs in a cost-effective manner, enabling dynamic scaling, burst processing for large datasets and geographic distribution of workloads. Together, these flexible deployment options ensure that *Magellon* can adapt to a wide range of user needs and institutional scales.

*Magellon*’s modular architecture enables distributed deployment, where each component can be installed on separate machines across a network. This distribution capability allows components to be strategically placed on hardware optimized for their specific requirements, such as GPU-accelerated nodes for computation-intensive plugins or high-memory servers for database operations. The system’s components automatically discover and communicate with each other through standardized APIs and message queuing, functioning cohesively as an integrated platform, regardless of physical location. This functionality is facilitated through the use of *Consul*, which is an open-source networking and service discovery tool for dynamic, distributed environments. *Consul* provides a registry where services can register themselves and discover other services, streamlining communication in microservice architectures.

This architecture provides both forward compatibility and scalability. Research facilities can manage their project uniformly through a single *Magellon* instance distributed across their infrastructure. The platform supports both vertical scaling (adding more CPUs, GPUs or storage to existing nodes) and horizontal scaling (incorporating additional computers or compute nodes into the system) without requiring architectural redesign. Moreover, with proper network configuration, *Magellon* enables geographic distribution of resources. Conceivably, a facility could maintain data storage in one location (*e.g.* Florida) while leveraging idle GPU resources at another institution or the cloud, creating a federated computing environment that maximizes resource utilization across sites.

There are two mechanisms for deployment, depending on the computational needs and expertise of the end user. For single workstation deployment, individual components have been containerized using *Docker*. A single *Docker*compose command can install all of the necessary dependencies onto a workstation of interest. For developers or on distributed systems, there is a CLI tool that was developed as a lightweight standalone executable. The CLI handles deployment, setup and management of both the *Magellon* core system and its plugins. Its design is platform-agnostic and has been tested on Windows, Linux and macOS. A single command, magellon install app, initiates the entire installation process. This command handles all the necessary environment setup and configuration, ensuring that users can get started with minimal effort and eliminating the need for manual configuration steps. The CLI simplifies the development process with the commands magellon development setup and magellon development load, which automatically configure the environment and load necessary dependencies. Together, these deployment tools provide a highly accessible and flexible entry point for users and developers, lowering barriers to adoption and promoting community-driven expansion of *Magellon*’s capabilities.

Plugin management is also handled with the *Magellon* CLI. Developers can create and deploy plugins using the commands magellon plugins create init, magellon plugins create myctf and magellon plugins deploy myctf. Likewise for end users, plugin discovery and installation are handled with the command magellon plugins list -a -s:author, which allows users to browse available plugins, while magellon plugins install myctf@latest enables seamless installation of specific plugins. The CLI makes it straightforward for users to expand *Magellon*’s functionality with minimal effort, without needing deep technical knowledge.

## 3. Methods

### 3.1. Data collection for demo

A small cryo-EM dataset was collected to demonstrate the functionality of *Magellon* for data import, management and processing. Mouse heavy-chain apoferritin from a plasmid gifted by Dr Masahide Kikkawa (Danev *et al.*, 2019 ▸) was expressed and purified as described previously (Peng *et al.*, 2023 ▸). UltraAuFoil R1.2/1.3 300 Mesh grids were plasma cleaned for 50 s at 7 W with 25% oxygen, 75% argon (Gatan Solarus 950). Cleaned grids were loaded onto Vitrobot Mark IV at 100% humidity (4°C). 4 µl of apoferritin was applied to the grids which were blotted for 1 s at zero force before being vitrified in liquid ethane. Data were collected on a Titan Krios microscope (ThermoFisher Scientific) operated at 300 kV with a 50 µm C2 aperture and 100 µm objective aperture and equipped with a DE Apollo direct detector. Images were acquired using *Leginon* (Suloway *et al.*, 2005 ▸). For high-magnification exposures, the Apollo was set in 8*k* × 8*k* super-resolution mode at a magnification of 59000× with a super-resolution pixel size of 0.395 Å. The movie frame rate was fixed at 60 frames per second.

## 4. Results

### 4.1. Deploying *Magellon* and importing data

As described above, *Magellon* can be installed and run using *Docker* or installed manually by setting up the required dependencies. Detailed instructions for both methods are available on the *Magellon* website (https://magellon.org) or the ReadMe file included with the source code. To help new users become familiar with *Magellon*, the code is accompanied by a test dataset that can be used to explore *Magellon* functionality. This small test dataset of vitrified apoferritin has been deposited in the Electron Microscopy Public Image Archive (EMPIAR) under the accession number EMPIAR-11254. The entry includes all 310 images associated with the dataset, including low-magnification images and 218 high-magnification exposures with accompanying movies.

To streamline data handling operations, we utilized Adaptors within *Magellon* to build a web tool for *Magellon* import/export, which enables users to import the test dataset seamlessly into the *Magellon Viewer*. We tested import on a variety of computers and platforms (Table 1 ▸). The test data includes both integrated micrographs and movies. During the import process, *Magellon* ingests associated metadata, creates image files and thumbnails for every image in PNG format, pre-calculates power spectra for every image and estimates the CTF for every image with a pixel size smaller than 5 Å per pixel. Since the distribution is containerized using *Docker*, the processing operations are platform-independent and the Linux binaries that *Magellon* wraps can be executed seamlessly on a variety of Linux distributions, Mac OS and Windows: essentially any OS for which *Docker* is compatible. The program *MotionCor2* serves as the default *Magellon* frame alignment plugin, so the frame alignment jobs will only execute on computers that are equipped with a compatible *Nvidia* GPU. However, the *Magellon* import process will proceed regardless of the presence of a GPU, enabled by the robust fault tolerance of *RabbitMQ*, which allows for the execution of jobs independently without the failure of one job propagating throughout the system.

We recommend that new users of *Magellon* become acquainted with the software by importing the test dataset and following the step-by-step demonstration included in our user guide. This demo provides an overview of *Magellon*’s key features and helps users understand how to integrate their own data into the system.

### 4.2. Interacting with *Magellon Viewer*

The first step for starting a session with *Magellon Viewer* is filling out a web based metadata form containing information about the user’s data. On completion, the user submits the import job, which triggers a series of customizable processes including metadata import, transfer of raw data to its final location, frame alignment and CTF estimation. Import can occur concurrently with data collection and the data processing occurs on-the-fly.

**Figure 4:**
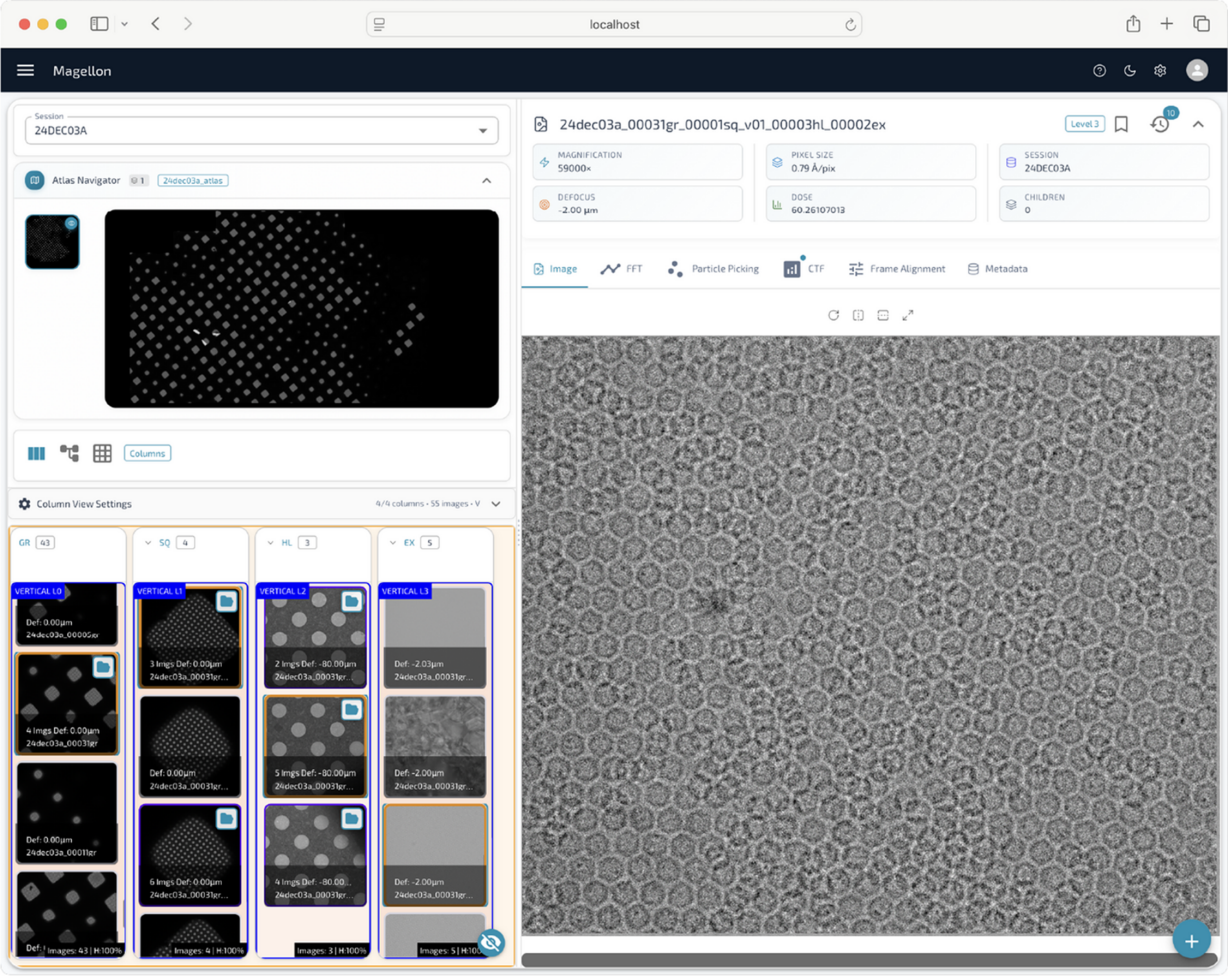
The *Magellon* viewer. The viewer shows atlases for a given session at the top left. A hierarchical image navigator is featured at the bottom left which allows users to navigate between lower magnification images and their higher magnification children. The center features an interactive viewer with associated metadata.

The *Magellon Viewer* graphical user interface was designed to maximize the visibility of the data and metadata. The interface is organized into four main sections: an atlas viewing section at the top left, a hierarchical image browser below the atlas section, a metadata display section on the upper right and a large interactive image viewer below the metadata section (Fig. 4 ▸). Users are presented with images representing the sample at every magnification used during data collection. Clicking on an atlas image reveals all the associated low-magnification images in the hierarchical browser, providing an overview of data acquisition at different scales. Colored bounding boxes highlight images that served as the basis for targeting higher-magnification acquisitions, visually guiding users through the imaging hierarchy.

When a user selects an image from the hierarchical browser, a full-resolution version is displayed in the interactive image viewer. This viewer offers a range of built-in tools, including the ability to display precomputed FFTs, a measuring tool for estimating distances or dimensions, and an interactive particle picker for manual or assisted particle selection. Key metadata, such as CTF estimation results, magnification and pixel size, are prominently displayed above the viewer for immediate reference. Full metadata records, including all recorded microscope parameters, camera settings and processing metadata are readily accessible through an expandable tab, enabling users to intuitively retrieve detailed information about each image and the corresponding acquisition conditions. This integrated, data-centric design ensures that users can efficiently navigate complex datasets, assess image quality and manage data collection strategies within a single unified platform.

### 4.3. Plugin development: *CTFFIND4* and *MotionCor2*

Two plugins have been developed for *Magellon* that serve as examples for future plugin developers. These two plugins carry out the initial image analysis processes that are universally performed on all cryo-EM data – frame alignment/dose-weighting and CTF estimation. Each type of image analysis process in *Magellon* is designated to a specific plugin category with defined metadata that are provided from *Magellon* to the plugin. Likewise, outputs are generated with the expected syntax, format and in an organized fashion. The inputs and outputs are managed by specific queues in the *Magellon* process manager controlled by *RabbitMQ*. Since inputs, outputs and metadata to and from the plugin are in a predefined format, the *Magellon* backend can handle queries, insert metadata into the *Magellon* database and generate organized output files. As *Magellon* continues to develop, more types will be developed (*e.g.* particle picking, image assessment *etc.*). This approach grants flexibility to developers, so that standardized inputs and outputs can be directed to any new algorithm, eliminating the need to rebuild or reconfigure the entire system.

The CTF estimation plugin was developed using the widely adopted *CTFFIND4* program (Rohou & Grigorieff, 2015 ▸). To manage task submission and execution, we established two *RabbitMQ* queues: an input queue for receiving processing requests and an output queue for returning results. The task is generated based on user-defined input parameters and submitted through the input queue. The input queue consumer retrieves each task and initiates the CTF estimation process, which comprises two main phases: CTF estimation and CTF evaluation. During the estimation phase, a command-line instruction for *CTFFIND4* is dynamically generated using the input parameters and executed via a subprocess call to the *CTFFIND4* binary. If an error occurs during execution, an error response is generated and returned; if successful, the outputs, including CTF parameters and diagnostic images, are captured for further evaluation. Following estimation, a secondary evaluation phase is conducted using quality metrics originally implemented in *Appion* (Sheth *et al.*, 2015 ▸). The outputs from *CTFFIND4* are subsequently analyzed, and all relevant metadata needed for CTF evaluation are collected and stored. Each request is assigned a unique directory, ensuring organized storage of output files and associated metadata. Outputs are formatted consistently across all plugins, then passed to the output queue for downstream processing. The result processor plugin subsequently retrieves and organizes the outputs, saving files to designated folders and recording metadata entries in the database. Outputs are classified into relevant categories – such as CTF or frame alignment – allowing seamless integration with the broader data management infrastructure. To ensure platform independence and facilitate deployment across diverse computing environments, both *CTFFIND4* and the associated CTF evaluation code were containerized using *Docker*.

The frame alignment plugin follows a similar architecture to the CTF plugin and was developed using the *MotionCor2* program (Zheng *et al.*, 2017 ▸). Like the CTF pipeline, frame alignment uses two dedicated *RabbitMQ* queues – one for input and one for output – to manage task submission and result handling. Because *MotionCor2* leverages GPU acceleration, special care was taken to ensure that all GPU drivers, libraries and dependencies were correctly installed and compatible with the version requirements of the software. On receiving a task from the input queue, the plugin dynamically generates the appropriate *MotionCor2* command using metadata retrieved from the *Magellon* database and executes the command via a subprocess. Log files, aligned micrographs and associated outputs generated during processing are systematically collected, organized into structured folders and registered within the database via the output queue. To promote platform independence and reproducibility, *MotionCor2* and its supporting components were containerized in a dedicated *Docker* image, so that the plugin runs consistently on any machine equipped with an appropriate *Nvidia* GPU.

Integration of all processing plugins into the *Magellon* core service in this manner ensures seamless operation within the broader platform. Task creation for plugins is automated by querying the necessary input metadata from the *Magellon* database and dispatching processing tasks to the appropriate input queues. The core service coordinates the execution of plugins by managing task scheduling, monitoring process status, and ensuring that outputs are appropriately stored and categorized. This modular and standardized architecture not only streamlines cryo-EM data processing but also facilitates extensibility, enabling straightforward incorporation of new algorithms and tools into the *Magellon* framework.

### 4.4. *Magellon*’s *Swagger* interface: a web based description of tools within *Magellon*

To enhance transparency, flexibility and ease of customization, *Magellon* provides users with direct access to backend services through an integrated *Swagger* interface. *Swagger* is an open-source tool for visualizing and interacting with *RESTful* APIs in a user-friendly, web based format. Rather than requiring users or developers to interact directly with the underlying codebase, *Magellon* exposes key backend functions through Swagger, which enables users to query data, launch processing jobs, manage configurations and automate workflows directly through a point-and-click web interface.

Users who install *Magellon* through the demo *Docker* containers can access the *Magellon*–*Swagger* interface by navigating to the website, https://localhost:8000/docs. Through this portal, users and developers can view detailed documentation of available backend functions, including descriptions of required input parameters and expected outputs. For example, users can retrieve specific dataset metadata, submit processing jobs or perform individual processing tasks. This design lowers the barrier for advanced usage and extension of *Magellon*, offering a straightforward pathway for users to explore backend capabilities and for developers to build custom features.

To introduce users to the *Swagger* interface, we implemented a standalone 2D lowpass filter function, which allows users to apply a lowpass filter to images already imported into *Magellon* or to new images uploaded by the user for on-the-fly filtering. Importantly, this example provides an accessible entry point for developers who want to begin building and testing their own image-processing algorithms within *Magellon*. Users can use this tool to selectively process individual images rather than the full dataset, showing how developers can quickly prototype and validate their routines in a lightweight, low-barrier environment. The lowpass filter plugin thus serves as a template for developers interested in contributing new functionality to the *Magellon* backend, demonstrating how custom processing functions can be exposed through the *Magellon* API and integrated into the larger workflow. Additional documentation and tutorials on how to use the *Swagger* interface, including examples for extending *Magellon* with new processing modules, are available on the *Magellon* website (https://magellon.org).

## 5. Discussion

New algorithms are continually being developed for cryo-EM from many different sources, each with its own dependencies. Integrating these new algorithms into a given processing pipeline represents a significant challenge for both developers and end users. A key aspect of *Magellon* is extensibility, and we have developed a plugin infrastructure that enables investigators to develop and deploy new utilities for cryo-EM data processing. We have developed two initial plugins including one for CTF estimation and one for frame alignment that serve as examples for future development.

*Magellon*’s architecture aligns with a broader shift in how cryo-EM data collection and processing are evolving. While current tools such as Thermo Fisher’s *CryoFlow*, *cryoSPARC Live*, and modules built around *Leginon* and *SerialEM* data collection software enable users to preprocess data and adjust acquisition parameters in real time, *Magellon* takes this concept significantly further. We envision a seamless, decoup­led workflow wherein data collection and analyses are no longer tied to the same physical location, but remain fully integrated operationally. *Magellon*’s microservice based deployment model and data management capabilities uniquely position it to enable this vision. For example, a researcher could initiate a data collection session at a remote facility through the *Magellon* web interface. As micrographs are acquired, they would immediately appear in the researcher’s browser – without any visible indication that behind the scenes, raw data are being (1) temporarily cached at the facility, (2) automatically transferred to high-performance cloud storage, (3) registered in a globally distributed *Magellon* database, (4) processed by dynamically provisioned cloud compute resources and (5) possibly being hosted at a geographically distant site, as the researcher visualizes the images through a web interface. The user remains shielded from the complexity of the underlying infrastructure, experiencing a unified and seamless workflow regardless of where the physical resources are located.

This location-transparent approach democratizes access to advanced cryo-EM capabilities, benefiting researchers in small laboratories or users of large research centers. Institutions without extensive local infrastructure could leverage cutting-edge microscopes and cloud based computational power without costly investments in hardware that rapidly becomes obsolete. Collaborators at different institutions could simultaneously view and analyze datasets in real time, thanks to *Magellon*’s ability to ensure a consistent and integrated view of project status and data processing. For facility managers, the architecture offers flexibility and scalability: computational resources can be dynamically scaled to meet demand, with operational costs tied directly to usage rather than fixed infrastructure capacity. Moreover, processing jobs could be routed to regions with lower-energy costs or available surplus compute capacity, improving both cost efficiency and environmental sustainability. *Magellon*’s current design, which separates data management, visualization and processing components, lays the critical foundation for this future, where physical resource constraints are minimized and scientific inquiry can proceed with unprecedented fluidity and reach.

While the current release of *Magellon* is mature in terms of the computational infrastructure and deployment, there remain several features that have not yet been implemented. For instance, the database and web frontend were built with the capacity to accommodate multiple users with role based and attribute based access control, but this has not been implemented in the current release. Likewise, the data processing workflow assumes the standard workflow for cryo-EM data processing (*e.g.* CTF estimation) and frame alignment. In future versions, users will be able to customize their workflows as more plugins such as particle pickers or micrograph assessors become available.

In the coming years, we anticipate that *Magellon* will evolve into a comprehensive cryo-EM data collection and viewing platform that will continue to integrate algorithms and software to enhance data acquisition efficiency. We plan to expand the plugin ecosystem, introducing additional tools for particle picking and image classification while simplifying capacity for user based development through a dedicated CLI, reusable SDK and robust documentation. A centralized plugin hub will facilitate sharing and collaboration among researchers, leading to new discoveries. The *Magellon* backend sets the foundation for future data collection tools that will leverage its database-backed processing infrastructure, enabling integration with *Magellon Viewer* for real time data visualization and assessment. Direct instrument integration will enable real-time control and data capture from the microscope and detectors, with the flexible infrastructure designed to accommodate both single particle as well as cryo-electron tomography data import, analysis and visualization. The current job management system will mature into a fully automated cryoEM data collection engine supporting advanced features that can distinguish appropriate regions for data collection and associated event triggers. Users will also benefit from a responsive supervision dashboard accessible from both desktop and mobile devices, permitting remote monitoring of collection sessions. Finally, we aim to streamline cloud deployment with one-click AWS implementation, enabling efficient cloud based data processing with intelligent resource management to optimize performance while minimizing costs.

## Supplementary Material

Test data for Magellon: https://doi.org/10.6019/EMPIAR-12604

## Figures and Tables

**Table 1:** Example import performance for the *Magellon* test dataset

Platform	Chip	GPU	RAM	Processing time (min)	Frame alignment
MacOS iMac	M1	None	16 G	19	None
Linux	Xeon W	GeForce RTX 4090	256 G	105	Yes
Linux	Xeon Silver	GeForce GTX 1080	64 G	359	Yes

## Data Availability

The apoferritin dataset used for testing is available on the Electron Microscopy Public Image Archive under accession code EMPIAR-11254. The code is available on GitHub (https://github.com/sstagg/Magellon.git) and the documentation is available on the *Magellon* website (https://www.magellon.org/).
